# Surfactant-Enriched Cross-Linked Scaffold as an Environmental and Manufacturing Feasible Approach to Boost Dissolution of Lipophilic Drugs

**DOI:** 10.3390/pharmaceutics17111387

**Published:** 2025-10-26

**Authors:** Abdelrahman Y. Sherif, Doaa Hasan Alshora, Mohamed A. Ibrahim

**Affiliations:** Department of Pharmaceutics, College of Pharmacy, King Saud University, Riyadh 11451, Saudi Arabia; dalahora@ksu.edu.sa

**Keywords:** surfactant-enriched cross-linked scaffold, manufacturing feasibility, environmental feasibility, pharmaceutical formulation, in vitro dissolution

## Abstract

**Background/Objectives**: The inherent low aqueous solubility of lipophilic drugs, belonging to Class II based on Biopharmaceutical classification system, negatively impacts their oral bioavailability. However, the manufacturing of pharmaceutical dosage forms for these drugs faces challenges related to environmental impact and production complexity. Herein, the surfactant-enriched cross-linked scaffold addresses the limitations of conventional approaches, such as the use of organic solvents, energy-intensive processing, and the demand for sophisticated equipment. **Methods**: Scaffold former (Pluronic F68) and scaffold trigger agent (propylene glycol) were used to prepare cross-linked scaffold loaded with candesartan cilexetil as a model for lipophilic drugs. Moreover, surfactants were selected based on the measured solubility to enhance formulation loading capacity. Design-Expert was used to study the impact of Tween 80, propylene glycol, and Pluronic F68 concentrations on the measured responses. In addition, in vitro dissolution study was implemented to investigate the drug release profile. The current approach was assessed against the limitations of conventional approach in terms of environmental and manufacturing feasibility. **Results**: The optimized formulation (59.27% Tween 80, 30% propylene glycol, 10.73% Pluronic F68) demonstrated a superior drug loading capacity (19.3 mg/g) and exhibited a solid-to-liquid phase transition at 35.5 °C. Moreover, it exhibited a rapid duration of solid-to-liquid transition within about 3 min. In vitro dissolution study revealed a remarkable enhancement in dissolution with 92.87% dissolution efficiency compared to 1.78% for the raw drug. **Conclusions**: Surfactant-enriched cross-linked scaffold reduced environmental impact by eliminating organic solvents usage and reducing energy consumption. Moreover, it offers significant manufacturing advantages through simplified production processing.

## 1. Introduction

The manufacturing of pharmaceutical dosage forms presents substantial challenges while developing oral dosage forms with minimal environmental impact and cost-effective production processes [1]. This issue is particularly prevalent during the preparation of pharmaceutical dosage forms containing poorly water-soluble drugs. This is ascribed to their mandatory demand for a drug delivery system able to enhance dissolution and oral bioavailability [2,3].

Extensive research over the past decades has led to the development of various delivery systems for enhancing bioavailability to address the challenges associated with these drugs. These drug delivery systems include solid dispersion [4,5], lipid-based formulations [6,7], polymeric nanoparticles [8,9], and complexation [10,11]. The production of solid dosage forms for the prepared drug delivery systems was typically achieved through various drying technologies. Among these methods, spray drying, lyophilization, and solvent evaporation are extensively used in the literature. However, the use of organic solvents and waste production limits the manufacturing and environmental feasibility of these processes [12].

Alternative solvent-free manufacturing approaches, such as microwave and hot melt extrusion, produce solid dosage forms. Nevertheless, these techniques present their own limitations, including extended processing times and the potential for drug degradation following exposure to thermal processing [13,14]. Consequently, the increasing demand for environmentally and manufacturing-feasible production processes underscores the crucial requirement for an alternative formulation approach.

In the present study, a surfactant-enriched cross-linked scaffold was presented to address the aforementioned challenges of the existing solidification approach. The innovation of surfactant-enriched cross-linked scaffold arises from its inherent solidification accompanied by an in vivo phase transition to a liquid state in response to physiological temperature. The combination of thermoresponsive polymers (Pluronic) and matrix-forming agent (propylene glycol) facilitates scaffold formation upon storage. Following oral administration, it undergoes rapid liquefaction in vivo following exposure to physiological temperature. Avoiding the use of extensive energy and organic solvents associated with other approaches is expected to minimize the negative environmental impact. On the other hand, the intrinsic solidification of the formulation following a simple mixing process of its ingredients eliminates the need for additional operations. This provides an outstanding advantage in terms of simplifying the manufacturing process.

The hydrophobic drug (candesartan cilexetil) was used as a model drug. It belongs to class II drugs based on the biopharmaceutical classification system, where their low solubility negatively impacts drug dissolution [15]. The reported low bioavailability of candesartan cilexetil (15%) necessitates the demand for a pharmaceutical approach to overcome this limitation [16].

The present study aimed to develop and optimize surfactant-enriched cross-linked scaffold loaded with candesartan cilexetil using Design-Expert software. A systematic assessment of formulation variables (Tween 80, propylene glycol, and Pluronic F68 concentrations) was conducted as the primary step to investigate their impact on the formulation response. The candesartan cilexetil-loaded surfactant-enriched cross-linked scaffold was characterized in terms of phase transition behavior and dissolution efficiency. This research seeks to establish surfactant-enriched cross-linked scaffold technology as a viable alternative to conventional solubility enhancement approaches.

## 2. Materials and Methods

### 2.1. Materials

Riyadh Pharma (Riyadh, Saudi Arabia) generously supplied candesartan cilexetil. Propylene glycol was obtained from Winlab Laboratory (Leicestershire, UK). Sigma-Aldrich (St. Louis, MO, USA) provided both ammonium formate and formic acid, which were used for the preparation of the aqueous component of the mobile phase. AppliChem Panreac (Darmstadt, Germany) provided acetonitrile, which was used as an organic component of the mobile phase. BDH (Poole, UK) and Techno Pharma Chem Haryana (Bahadurgarh, India) supplied tween-20 and tween-80, respectively. BASF (Ludwigshafen, Germany) supplied Kolliphor EL. Merck (Darmstadt, Germany) provided Tween-60, tween-85, and span-80 (S-80). Nicole Chemical Co. (Tokyo, Japan) supplied hydrogenated castor oil (HCO-10, 30, and 60).

### 2.2. Selection of Surfactant

The surfactant for the proposed formulation was selected based on two critical criteria. Firstly, the chosen surfactant should be miscible with propylene glycol (scaffold-former material) to avoid instability issues during storage. Secondly, the surfactant with the highest solubilization power should be selected to achieve higher drug loading, which increases the formulation’s loading capacity.

#### 2.2.1. Miscibility of Propylene Glycol

The miscibility of propylene glycol was screened in various types of surfactants (Tween 80, Tween 85, Tween 60, Tween 20, Span 80, Span 20, HCO-60, HCO-30, HCO-10, and Kolliphor EL). A mixture of propylene glycol and surfactant in a ratio of 1:1 was prepared and mixed for 1 day at 1000 rpm at 23 ± 2 °C. After that, the mixture was stored for 1 day and its physical appearance was visually checked to assess miscibility.

#### 2.2.2. Solubility of Candesartan Cilexetil

An excess amount of candesartan cilexetil was added to a homogeneous blend of propylene glycol and surfactant. After that, the mixture was stirred using a magnetic stirrer at 1000 rpm for 1 day at 23 ± 2 °C to determine the saturation solubility of the drug. Within the next day, the mixture was centrifuged for 15 min at 13,000 rpm to precipitate the undissolved powder of candesartan cilexetil to ensure accurate estimation of drug solubility. An accurately weighed amount from the supernatant was mixed with 1.8 mL of organic solvent (acetonitrile) and subjected to sonication for 15 min to guarantee complete extraction of the drug. Drug concentration was determined by comparing the sample with a calibration curve developed using a UPLC method.

### 2.3. Experimental Design

An optimal mixture design was employed to optimize surfactant-enriched cross-linked scaffold using Design-Expert 13 software (Stat-Ease, Inc., Minneapolis, MN, USA). The influence of three mixture components on the measured responses was investigated in the following ranges: Tween 80 concentration (30–65%), propylene glycol concentration (30–65%), and Pluronic F68 concentration (5–15%). An I-optimal approach was implemented owing to its expected superior and accurate prediction capabilities. Table 1 presents the suggested design, which comprises 17 experimental runs, including seven model points, five lack-of-fit points, and five replicate points. Drug loading (mg/g), onset of solid-to-liquid transition (°C), and duration of solid-to-liquid transition (sec) were selected as variable responses. Response surface methodology was employed to identify optimal formulation conditions and understand the relationship between mixture components and responses. Model selection for each response was based on the sequential model sum of squares, where the highest-order polynomial with significant additional terms and a non-aliased model was selected. Model fitness was evaluated using the coefficient of determination (R^2^), adjusted R^2^, predicted R^2^, and adequate precision ratio. Models with R^2^ > 0.80, adequate precision (defined as >4), and a non-significant lack of fit were deemed acceptable for optimization purposes.

### 2.4. Preparation of Optimum Surfactant-Enriched Cross-Linked Scaffold

The ingredients mentioned in Table 1 were placed in a glass beaker, and the mixture was transferred to an incubator set at 50.0 ± 0.5 °C until Pluronic F68 was completely dissolved. The prepared surfactant-enriched cross-linked scaffold was placed in a 5 mL glass beaker to measure drug solubility. Moreover, 1.0 ± 0.1 g of the prepared surfactant-enriched cross-linked scaffold was transferred to a glass test tube and left to solidify in the refrigerator before measuring the onset of solid-to-liquid transition and the duration of solid-to-liquid transition. To prepare the drug-loaded formulation, a loading concentration of 15.4 mg/g (80% of the maximum drug loading capacity) was used to prepare candesartan cilexetil-loaded surfactant-enriched cross-linked scaffold.

### 2.5. Drug Loading

Drug loading was calculated using the same procedure mentioned in the solubility study for the selection of surfactant presented in Section 2.2.2.

### 2.6. Onset of Solid-to-Liquid Transition

The following procedure was applied to determine the temperature at which the surfactant-enriched cross-linked scaffold underwent a phase transformation from a solid to a liquid at atmospheric pressure (1 atm). Briefly, a temperature-controlled environment was maintained using a water bath with a temperature of 28 ± 0.5 °C at the beginning of the experiment. After that, test tubes containing surfactant-enriched cross-linked scaffolds were positioned in a rack and submerged in the water bath to achieve equilibrium. Subsequently, the rack was removed from the water bath, and the test tubes were visually inspected. The onset of solid-to-liquid transition was recorded for a surfactant-enriched cross-linked scaffold, which showed a transition to a liquid state. The water bath temperature increased by 0.5 ± 0.1 °C, and the above procedures were repeated until the onset of solid-to-liquid transition for all surfactant-enriched cross-linked scaffolds was measured.

### 2.7. Duration of Solid-to-Liquid Transition

The time required for a surfactant-enriched cross-linked scaffold to undergo a complete phase transition from a solid to a liquid state under physiological conditions was assessed using the following procedure. The water bath was adjusted to body temperature (37 ± 0.1 °C) at the beginning of the experiment. The duration of solid-to-liquid transition for each formulation was estimated separately at atmospheric pressure (1 atm). The transition duration was quantified by recording the elapsed time from the initial immersion of the tube containing the surfactant-enriched, cross-linked scaffold into the bath until it was completely converted to a liquid state.

### 2.8. Selection of Optimized Formulation

The optimization criteria were established based on therapeutic requirements and practical considerations for the surfactant-enriched cross-linked scaffold. Maximizing drug loading was considered within the studied range to reduce the total dosage required. This is important to reduce the final number of pills and fill the predetermined dose within one capsule. The onset of solid-to-liquid transition was targeted at 36 °C to ensure conversion to the liquid state at physiological temperature while maintaining a safe margin during storage conditions. On the other hand, the duration of solid-to-liquid transition parameter was set within a studied range, as it has a non-critical impact on the surfactant-enriched cross-linked scaffold. This is ascribed to the conversion of all surfactant-enriched cross-linked scaffolds to liquid within less than 4 min. The optimized surfactant-enriched cross-linked scaffold was prepared, and the observed values were compared against the confidence interval to validate the design.

### 2.9. Solid-to-Liquid Transition Assessment

The solid-to-liquid transition capability of a surfactant-enriched cross-linked scaffold was evaluated to ensure a hypothetical transition to a liquid state in vivo. To achieve this, a beaker filled with preheated water (100 mL) was used. The capsule-filled with surfactant-enriched, cross-linked scaffold was embedded within the sinker to ensure complete exposure to physiological temperatures. Images of the capsule-filled with surfactant-enriched cross-linked scaffold were taken at the beginning and after incubation for documentation.

### 2.10. In Vitro Dissolution

Dissolution apparatus with a paddle (LOGAN Inst. Corp., Somerset, NJ, USA) was utilized to compare the dissolution performance of candesartan cilexetil from raw drug and marketed tablet against the optimized surfactant-enriched cross-linked scaffold. Within a hard gelatin capsule, 8 mg of candesartan cilexetil and an equivalent amount of surfactant-enriched cross-linked scaffold formulation were placed to prepare a pharmaceutical dosage form. To guarantee capsule immersion, it was placed encircled with wire before the experiment began. Dissolution media consisting of phosphate buffer (pH 6.8, 900 mL) were placed in a vessel that was preheated to 37 ± 0.5 °C before the dissolution study. Surfactant-enriched cross-linked scaffold formulations surrounded by a sinker were placed in each vessel, whereas the paddle speed was rotated at 50 rpm. Samples were withdrawn during the predetermined intervals using a syringe connected to a filter, and the drug concentration was estimated using the UPLC method.

## 3. Results and Discussion

### 3.1. Mechanistic Solid-to-Liquid Transition

**Figure 1:**
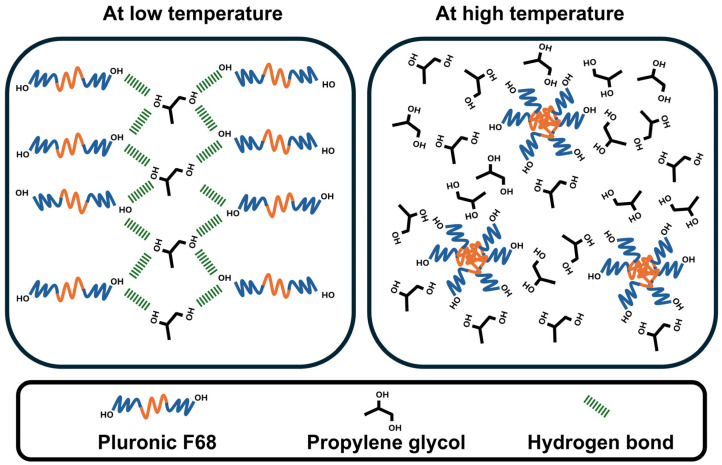
The sketch shows the possible mechanistic transition of a surfactant-enriched cross-linked scaffold depending on the temperature from a solid (**left**) to a liquid (**right**) state and vice versa.

Pluronic F68 was used in the present study as a thermoresponsive polymer based on its ability to rearrange its monomeric units in response to temperature. Moreover, an organic compound containing two hydroxyl groups (propylene glycol) was selected based on a preliminary study. Initially, a cross-linked scaffold was prepared using the aforementioned two agents (Pluronic F68 and propylene glycol) to prepare a drug delivery system. This system has reversible solid-to-liquid and liquid-to-solid transition behavior. Figure 1 shows the expected change in Pluronic F68 arrangement during cooling and heating, which is responsible for this transition. At low temperature, cross-linking hydrogen bonding between Pluronic F68 and propylene glycol resulted in a transition to a solid scaffold. On the other hand, increasing temperature breaks the formed hydrogen bonds, facilitates the micellar arrangement of Pluronic F68, and triggers the liquification of the scaffold.

Propylene glycol showed low capability to dissolve candesartan cilexetil with a value of 0.97 mg/g. The inclusion of Pluronic F68 at a 20% *w*/*w* concentration was only able to increase candesartan cilexetil solubility to 4.97 mg/g. Unfortunately, the expected total dosage was ≈1.6 g from the prepared cross-linked scaffold, which needs to be filled within two capsules. Therefore, a surfactant-enriched cross-linked scaffold was proposed here to enhance its acceptability by patients and reduce the number of capsules needed to be administered. For this purpose, the screening criteria in the next section were applied to select a surfactant that is miscible with cross-linked scaffold components and exhibits a superior solubilization power for candesartan cilexetil.

### 3.2. Selection of Surfactant

The miscibility of propylene glycol with the chosen surfactants was evaluated as the primary step for surfactant selection, and the results are presented in Table 2. Visual assessment revealed that Tween 80, Tween 85, Tween 60, Tween 20, HCO-60, HCO-30, and Kolliphor EL were completely miscible with propylene glycol. However, Span 80, Span 20, and HCO-10 failed to form a homogenous mixture when mixed with propylene glycol. Therefore, these surfactants were excluded from the study to avoid instability issues expected to arise from the storage of the formulation.

The solubility test was implemented as the second step in selecting a surfactant to prepare a surfactant-enriched cross-linked scaffold. The current results showed that Tween 80 demonstrated the highest solubilization capacity for candesartan cilexetil with a value of 12.57 ± 0.14 mg/g. Thus, Tween 80 was selected as the optimal surfactant for further formulation development due to its miscibility with propylene glycol and its superior solubilization capacity for candesartan cilexetil. This is expected to enhance the pharmaceutical and clinical applicability of the surfactant-enriched cross-linked scaffold by reducing the number of pills required for administration.

### 3.3. Model Validation for Studied Responses

**Figure 2:**
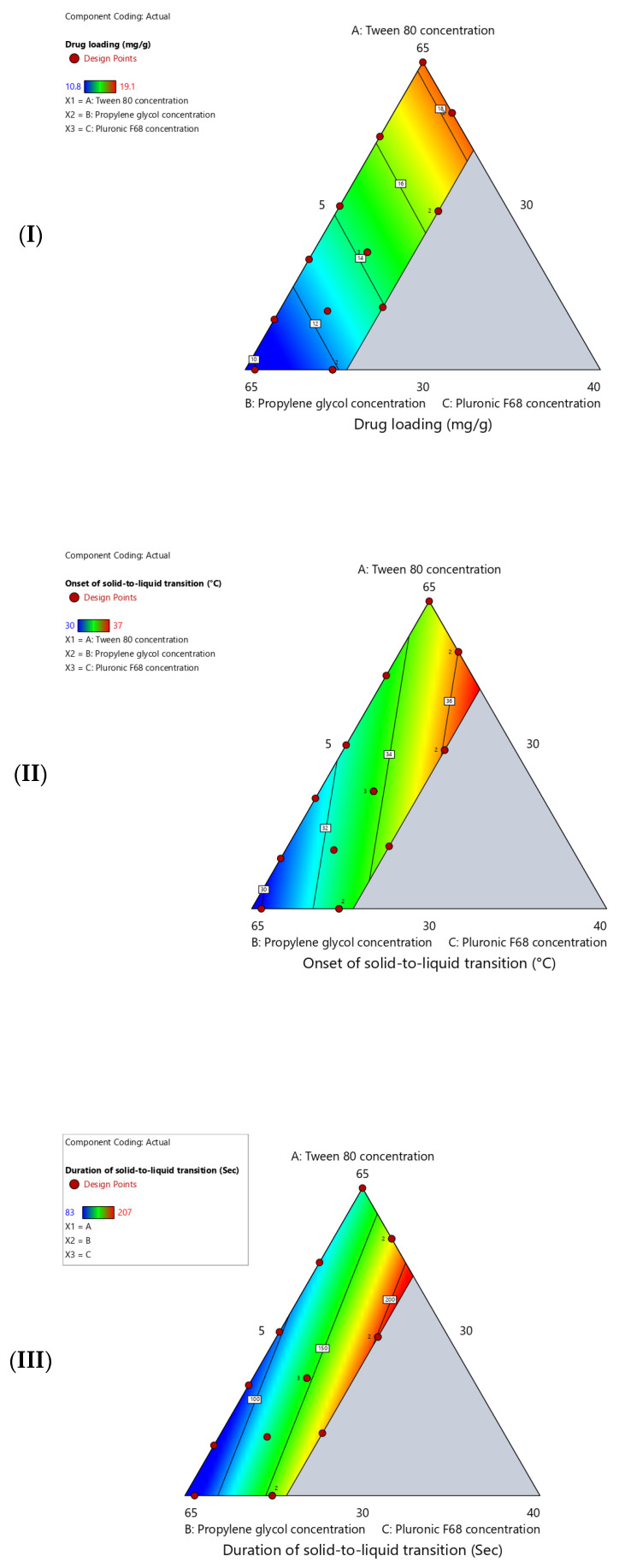
Contour plot shows the influence of (A) Tween 80 concentration, (B) propylene glycol concentration, and (C) Pluronic F68 concentration on (**I**) drug loading (mg/g), (**II**) onset of solid-to-liquid transition (°C), and (**III**) duration of solid-to-liquid transition (Sec).

The measured values for the studied response (drug loading, onset of solid-to-liquid transition, and duration of solid-to-liquid transition) of the suggested surfactant-enriched cross-linked scaffold are presented in Table 3. The impact of mixture components (Tween 80, propylene glycol, and Pluronic F68) on responses is statistically analyzed using Design-Expert software. Table 4 shows the estimated parameters for the selected linear model for all responses. All three responses demonstrate predictive capability with R^2^ values ranging from 0.8418 to 0.9576 with a highly significant *p*-value (*p* < 0.0001). Furthermore, the close agreement between R^2^ and adjusted R^2^ values for all responses confirms that the models are not overfitted. Additionally, excellent predictive capability for the selected model was confirmed by the calculated predicted R^2^, which ranged from 0.7324 to 0.9332. In addition, the adequate precision values (>4.0) indicate that the selected models are acceptable. The significance of selected models is confirmed by the estimated high F-values (*p* < 0.0001). This confirms that the estimated linear relationship between components and responses is not due to chance. Moreover, the observed non-significant (*p* > 0.05) lack-of-fit tests confirmed the reliability of the established linear relationship. Figure 2 shows a contour plot that represents the influence of three component concentrations on the parameters of surfactant-enriched cross-linked scaffolds (drug loading, onset of solid-to-liquid transition, and duration of solid-to-liquid transition).

### 3.4. Drug Loading Response Analysis

**Figure 3:**
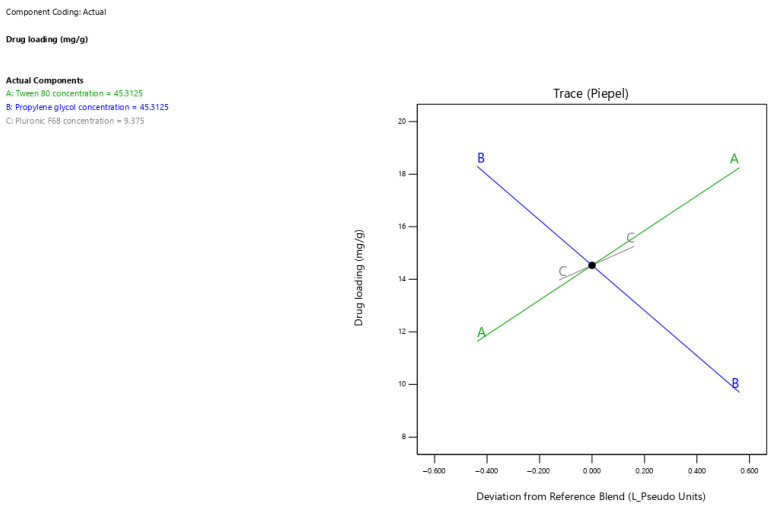
The trace plot illustrates the individual effects of mixture components on the drug loading response.

The current results showed that surfactant-enriched cross-linked scaffolds exhibited drug loading values ranging from 10.8 to 19.1 mg/g (Table 3). Figure 3 presents the trace plot illustrating the effects of Tween 80, propylene glycol, and Pluronic F68 on drug loading. The positive slopes in the trace plot for tween 80 and pluronic F68 concentrations indicate their positive influence on drug loading. In contrast, the negative slope for propylene glycol concentration indicates its adverse influence on drug loading. The statistical analysis presented in Table 5 indicates that all formulation components significantly affect the drug loading capacity (*p* < 0.05). The estimated effect values 6.61 and 1.28 for Tween 80 and Pluronic F68 concentrations indicate the predominant positive impact of Tween 80. In contrast, propylene glycol demonstrated a pronounced adverse effect on drug loading, as indicated by its high magnitude of negative value (−8.60). The drug loading capacity for surfactant-enriched cross-linked scaffolds at any level of the selected range could be estimated using Equation (1):

Drug loading = 0.26 × Tween 80 concentration + 0.01 × Propylene glycol concentration + 0.26 × Pluronic F68 concentration(1)

The observed significant positive impact of Tween-80 agrees with the current solubility study, which revealed its superior solubilization capacity. This could be attributed to the formation of favorable bonds between the functional groups present in Tween 80 and candesartan cilexetil. The reported hydrogen-bonding capability of the latter is expected to enhance solubility in Tween 80 through interactions with its free hydroxyl groups [17]. In addition, the presence of oleic acid oil within Tween-80 facilitated the solubilization of candesartan cilexetil through the reported hydrophobic interaction. The observed high solubilization power of Tween 80 agrees with previously published studies in the literature [18]. Additionally, Pluronic F68 exhibited a significant but inferior effect on the solubility of candesartan cilexetil compared to Tween-80. The presence of polypropylene oxide chains within the triblock structure of Pluronic F68 provides a lipophilic environment for the solubilization of candesartan cilexetil [19]. In contrast, propylene glycol showed a significant negative impact on the solubility of candesartan cilexetil, aligning with the preliminary screening study. Although propylene glycol possesses two hydroxyl groups capable of hydrogen bonding with candesartan cilexetil, its overall solubilization capacity for this lipophilic drug is limited. The predominantly hydrophilic character of propylene glycol results in insufficient hydrophobic domains to accommodate the extensive lipophilic regions of candesartan cilexetil (biphenyl tetrazole moiety and cyclohexyl ester groups). Furthermore, the relatively high polarity of propylene glycol (dielectric constant ≈ 32) creates an unfavorable environment for lipophilic drug solubilization [20].

### 3.5. Onset of Solid-to-Liquid Transition

**Figure 4:**
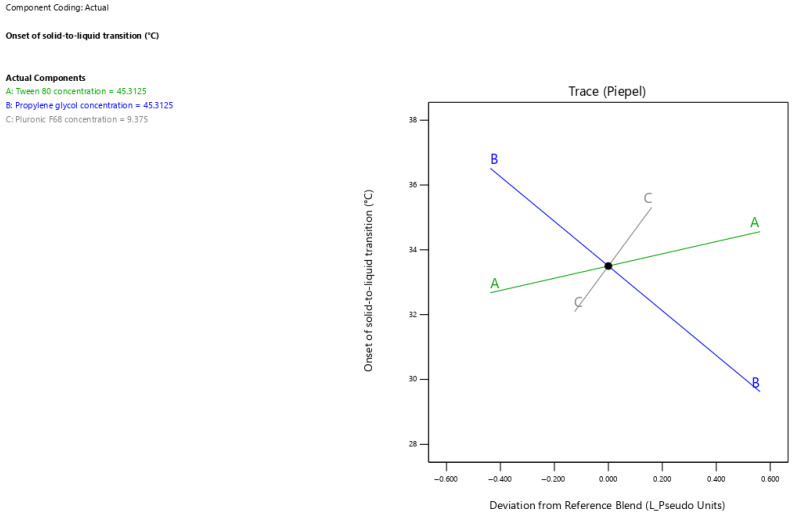
Trace plot showing the individual impacts of mixture components on the onset of solid-to-liquid transition response.

The current results showed that surfactant-enriched cross-linked scaffold exhibited onset of solid-to-liquid transition values ranging from 30.5 to 37 °C (Table 3). Figure 4 presents the trace plot illustrating the effects of Tween 80, propylene glycol, and Pluronic F68 on the onset of solid-to-liquid transition. The positive slopes in the trace plot for Tween 80 and Pluronic F68 concentrations indicate their positive influence on the onset of solid-to-liquid transition. On the other hand, the negative slope for propylene glycol concentration indicates its adverse influence on the onset of solid-to-liquid transition. The statistical analysis presented in Table 5 shows that all surfactant-enriched cross-linked scaffold components significantly affected the onset of solid-to-liquid transition (*p* < 0.05). The estimated effect values of 1.89 and 3.22 for Tween 80 and Pluronic F68 concentrations, respectively, indicate a predominant positive impact of Pluronic F68. In contrast, propylene glycol demonstrated a pronounced adverse effect on the onset of solid-to-liquid transition, as indicated by its high negative effect value (−6.89). The onset of solid-to-liquid transition for surfactant-enriched cross-linked scaffolds at any level of the selected range could be estimated using Equation (2):

onset of solid-to-liquid transition = 0.38 × Tween 80 concentration + 0.23 × Propylene glycol concentration + 0.63 × Pluronic F68 concentration (2)

The increase in the onset of solid-to-liquid transition with increasing Tween 80 concentration can be attributed to the presence of a long polyethylene oxide chain within its structure. This could result in its intercalation within the cross-linked structure of the scaffold. Moreover, the presence of non-covalent bond interactions between the polyethylene oxide chains of Tween-80 and Pluronic F68 could require more energy to disrupt. Therefore, it is necessary to increase the temperature of the surfactant-enriched cross-linked scaffold for the disassembly of the formed scaffold. Although Pluronic F68 showed a similar positive impact to Tween-80, an additional mechanism may augment its pronounced effect on the onset of solid-to-liquid transition. An increase in Pluronic F68 concentration augments the formation of cross-link bonds within the prepared scaffold. This increases the temperature required for the complete solid-to-liquid transition in a surfactant-enriched cross-linked scaffold containing a higher concentration of Pluronic F68. The present outcome is in harmony with the literature revealing a direct correlation between polymer concentration and intermolecular bonding [21]. On the contrary, the increment in propylene glycol concentration altered the integrity of the formed scaffold matrix, which significantly reduced the onset of solid-to-liquid transition. The abundance of small molecular structures facilitated the breakage of the hydrogen bonds within the scaffold, unlike those enriched with a longer chain of Pluronic F68.

### 3.6. Duration of Solid-to-Liquid Transition

**Figure 5:**
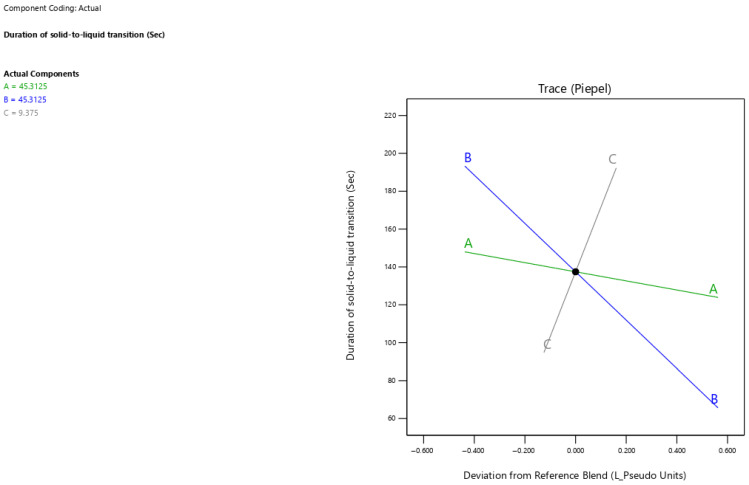
Trace plot showing the individual impacts of mixture components on the duration of solid-to-liquid transition response.

The current results showed that surfactant-enriched cross-linked scaffold formulations exhibited solid-to-liquid transition duration values ranging from 83 to 207 s (Table 3). Figure 5 presents the trace plot illustrating the effects of Tween 80, propylene glycol, and Pluronic F68 on the duration of solid-to-liquid transition. The positive slope in the trace plot for Pluronic F68 concentration indicates its positive influence on the duration of solid-to-liquid transition. In contrast, the negative slopes for Tween 80 and propylene glycol concentrations indicate their adverse influence on the duration of solid-to-liquid transition. The statistical analysis presented in Table 5 reveals that propylene glycol and Pluronic F68 significantly impacted the duration of solid-to-liquid transition (*p* < 0.001), whereas Tween 80 showed no significant effect (*p* > 0.05). The estimated effect values −107.93 and 97.54 for propylene glycol and Pluronic F68 demonstrate opposing but equally strong impacts on the duration of solid-to-liquid transition. The duration of solid-to-liquid transition for surfactant-enriched cross-linked scaffolds at any level of the selected range could be estimated using Equation (3):

Duration of solid-to-liquid transition = 1.29 × Tween 80 concentration − 0.37 × Propylene glycol concentration + 10.21 × Pluronic F68 concentration (3)

The direct correlation between Pluronic F68 concentration and duration for the solid-to-liquid transition could be ascribed to the abundance of strong cross-linked interactions with the scaffold [22]. Thus, the required duration for breaking cross-linked interactions within the scaffold is increased. On the other hand, propylene glycol produced a destructive impact on the abundance of strong cross-linked interactions with the scaffold. Therefore, the time needed for the formulation to become liquid and break the cross-linked interactions decreased as the propylene glycol concentration increased.

### 3.7. Selection of Optimized Formulation

**Figure 6:**
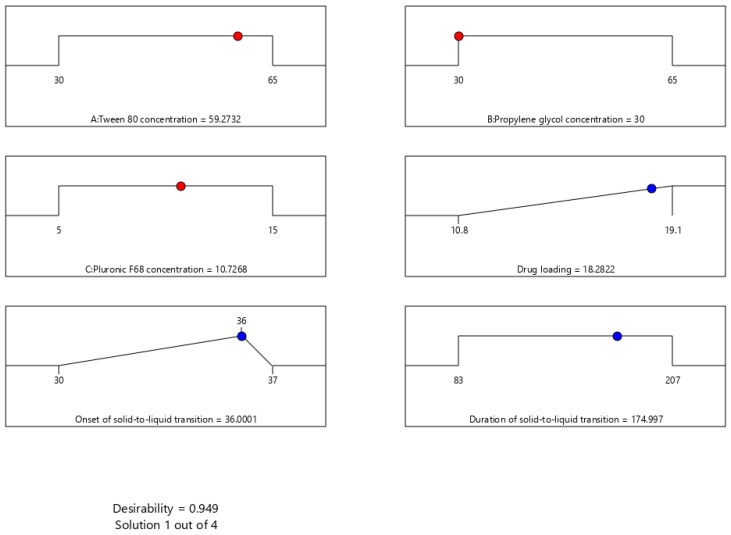
Ramp plots displaying the optimal levels of surfactant-enriched cross-linked scaffold formulation components based on desirability function optimization. The plots show the optimized concentrations of Tween 80 (59.27% *w*/*w*), propylene glycol (30% *w*/*w*), and Pluronic F68 (10.73% *w*/*w*). This is expected to maximize drug loading (18.28 mg/g), target the set onset of solid-to-liquid transition (36 °C), and achieve a duration of solid-to-liquid transition within the set range (174.997 s).

The criteria used to suggest an optimized formulation were based on maximizing drug loading capacity. This criterion reduces the expected frequency of administration due to the anticipated reduction in the total formulation dosage. However, the onset of solid-to-liquid transition was suggested to target 36 °C, which provides a protection boundary against formulation liquification during storage. Additionally, selecting a target temperature lower than the physiological body temperature ensures solid-to-liquid conversion after administering the dosage form. However, the duration of solid-to-liquid transition was not included in the suggested criteria because the acceptable limit for all measured values across the design space was met. Figure 6 shows the desirability of the optimized formulation (0.949), along with the levels of its components and the corresponding expected response values. The suggested formulation mixture consists of Tween-80, propylene glycol, and Pluronic F68 with concentration values of 59.27, 30, and 10.73% *w*/*w*, respectively. This formulation is expected to have a drug loading of 18.28 mg/g, an onset of solid-to-liquid transition of 36 °C, and a duration of solid-to-liquid transition of 175 s, respectively.

The recommended formulation was prepared to ensure the validity of the current design in predicting response values across the considered range. The measured results values of three independent formulations, along with the low and high 95% prediction intervals, were presented in Table 6. The optimized formulation showed a high loading capacity with a value of 19.3 mg/g and exhibited a threshold solid-to-liquid at 35.5 °C. Moreover, it exhibited a duration of solid-to-liquid transition within 188.0 s. The high prediction power of the current model was confirmed by the inclusion of all experimental mean values within the 95% prediction interval limit.

### 3.8. Solid-to-Liquid Transition Assessment

**Figure 7:**
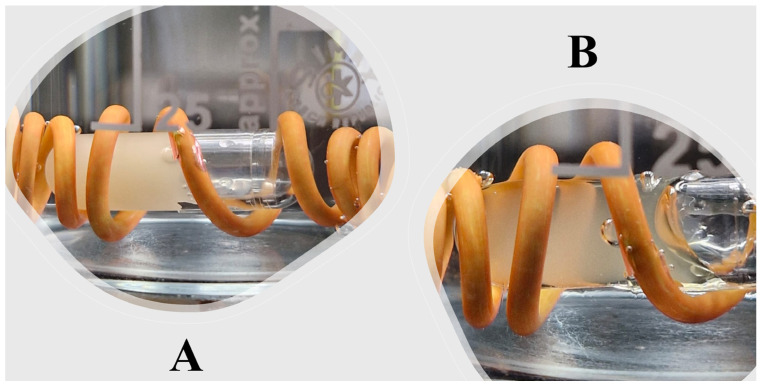
Surfactant-enriched cross-linked scaffold during physiological assessment that presents at (**A**) solid and (**B**) liquid state at the beginning and after incubation, respectively.

Before starting the solid-to-liquid transition assessment, the surfactant-enriched cross-linked scaffold was filled and left to solidify. After that, the capsule filled with solid surfactant-enriched cross-linked scaffold was surrounded with a wire and immersed in preheated water. Figure 7A shows that the surfactant-enriched cross-linked scaffold exists in a solid state. However, the physical appearance of the surfactant-enriched cross-linked scaffold became liquid after exposure to physiological temperature (Figure 7B). The present study validates the pharmaceutical applicability of a surfactant-enriched cross-linked scaffold for patient administration. The absence of drug precipitation and complete transition to the liquid state needs further assessment to ensure enhanced drug dissolution once exposed to intestinal simulated media. Therefore, an in vitro dissolution study was performed as the final step for clinical applicability of a surfactant-enriched cross-linked scaffold.

### 3.9. In Vitro Dissolution Study

**Figure 8:**
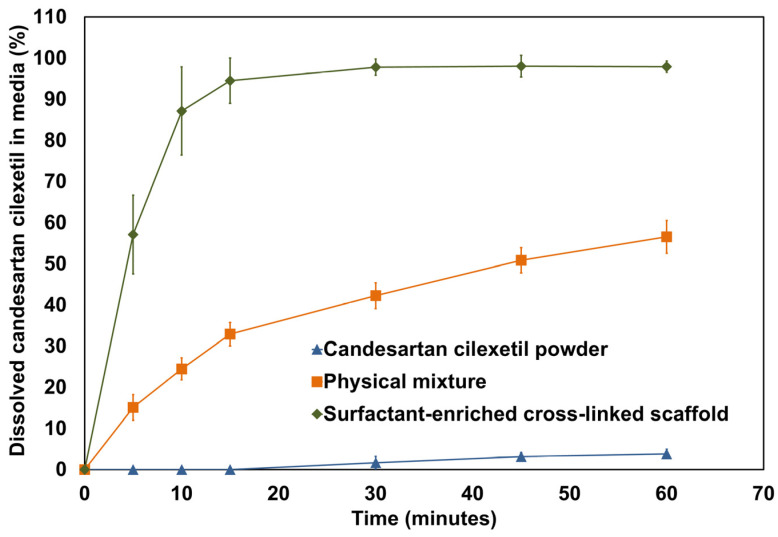
In vitro dissolution profile of candesartan cilexetil powder, surfactant-enriched cross-linked scaffold, and physical mixture of its components. Data are expressed as mean ± SD (n = 3).

The calculated percentage of dissolved candesartan cilexetil from capsules filled with powder, physical mixture, and surfactant-enriched cross-linked scaffold formulations in the dissolution media was plotted and shown in Figure 8. Moreover, Table 7 presents the estimated initial dissolution rate and dissolution efficiency for the tested agents. The initial dissolution rate was estimated for the first 15 min to provide a clear impression of the impact of formulation type on dissolution. The results showed that candesartan cilexetil powder exhibited no drug release at the beginning of the study, which is consistent with its reported hydrophobic properties. Although a physical mixture enhanced drug dissolution, a surfactant-enriched cross-linked scaffold increased the initial dissolution rate of candesartan cilexetil by approximately 3-fold. Furthermore, the overall enhancement in candesartan cilexetil dissolution was assumed from dissolution efficiency. The results revealed a significant ability of the surfactant-enriched cross-linked scaffold to increase the dissolution efficiency of untreated candesartan cilexetil from 1.78 to 92.87%.

The observed enhancement in candesartan cilexetil using surfactant-enriched cross-linked scaffold could be ascribed to the following mechanisms. The observed incredible enhancement in the percentage of dissolved candesartan cilexetil (57.2%) at 5 min reflects the rapid transition of the surfactant-enriched cross-linked scaffold to the liquid state. The present results indicate the quick responsiveness of a surfactant-enriched cross-linked scaffold to body temperature. Moreover, 97.8% of the candesartan cilexetil was dissolved within 30 min. This provides an additional advantage regarding the reported limitation of drug entrapment within the matrix of traditional solid dosage forms. The observed complete drug solubilization within 30 min can be attributed to the molecular dispersion of candesartan cilexetil within the solubilized state of the cross-linked scaffold. This provides strong indirect evidence regarding the molecular-level dispersion of the drug and its existence in an amorphous state. The current data suggested that a prepared formulation could significantly enhance candesartan bioavailability [23,24].

### 3.10. Environmental Feasibility of the Current Approach

The environmental impact assessment of the surfactant-enriched cross-linked scaffold demonstrates substantial advantages over conventional bioavailability enhancement approaches. The elimination of organic solvents represents a significant ecological breakthrough. This is ascribed to its ability to avoid volatile organic compound emissions, air pollution, and hazardous waste disposal requirements associated with spray drying, lyophilization, and solvent evaporation methods [25]. A reduction in energy consumption compared to traditional methods, which use energy-intensive processes, compared to mild heating (50 °C for 1 h) while preparing surfactant-enriched cross-linked scaffold [26]. This reduction in energy demand translates to a decreased carbon footprint and lower greenhouse gas emissions throughout the manufacturing lifecycle [27]. Water resource conservation is achieved through the elimination of extensive washing and purification steps required in conventional methods. Simplified processing also eliminates transportation requirements for organic solvents and reduces the use of packaging materials for hazardous chemicals [28].

### 3.11. Manufacturing Feasibility of the Current Approach

The manufacturing feasibility assessment of the surfactant-enriched cross-linked scaffold demonstrates exceptional advantages over conventional bioavailability enhancement approaches across multiple industrial parameters. The simplified preparation process requires only standard mixing equipment and temperature-controlled incubators capable of maintaining a temperature of 50 °C. This eliminates the need for sophisticated and expensive machinery, such as spray dryers, lyophilizers, or hot melt extruders, which require substantial capital investments and specialized maintenance. Processing time is dramatically reduced to 2–3 h compared to 24–72 h for conventional methods, enabling higher throughput and improved manufacturing flexibility [29]. The absence of complex drying, milling, or solvent recovery steps significantly reduces operational costs and the need for quality control. Scalability is enhanced through a straightforward mixing and heating process that maintains consistent quality parameters across batch sizes. This eliminates the heat and mass transfer limitations encountered in traditional approaches. The elimination of residual solvent testing reduces analytical requirements by 60–70%, while the inherent process robustness minimizes batch failure risks and associated costs [30]. Furthermore, the room temperature stability of the final product eliminates the need for cold storage, and the simple process parameters facilitate regulatory compliance and validation procedures, positioning the surfactant-enriched cross-linked scaffold as a manufacturing-feasible alternative that combines operational simplicity with superior pharmaceutical performance.

## 4. Conclusions

The present study successfully developed and optimized a novel surfactant-enriched cross-linked scaffold that addresses critical limitations of conventional bioavailability enhancement approaches. The optimized surfactant-enriched cross-linked scaffold formulation achieved a remarkable drug loading capacity of 19.3 mg/g. The extraordinary enhancement in dissolution efficiency from 1.78% to 92.87% demonstrates the superior pharmaceutical performance of the surfactant-enriched cross-linked scaffold approach. It offers substantial manufacturing advantages through its simplified preparation process, which eliminates the need for organic solvents, energy-intensive operations, and complex equipment typically required for other methods. This approach aligns with increasing demands for sustainable pharmaceutical manufacturing processes while achieving superior therapeutic outcomes. The ability to produce high-performance formulations through simple mixing and inherent solidification, without the need for additional procedures, represents a paradigm shift toward environmentally responsible drug development. Further in vivo pharmacokinetic and pharmacodynamic study is required to confirm the bioavailability enhancement ability of the current approach.

## Figures and Tables

**Table 1:** Experimental design for surfactant-enriched cross-linked scaffold suggested by Design-Expert software.

Run	Tween 80 Concentration	Propylene Glycol Concentration	Pluronic F68 Concentration
1	42.6	52.4	5.0
2	37.1	47.9	15.0
3	43.4	46.3	10.3
4	30.0	57.0	13.6
5	30.0	65.0	5.0
6	48.1	36.9	15.0
7	65.0	30.0	5.0
8	43.4	46.3	10.3
9	35.7	59.3	5.0
10	36.7	53.6	9.8
11	48.6	46.4	5.0
12	59.2	30.0	10.8
13	48.1	36.9	15.0
14	43.4	46.3	10.3
15	56.5	38.5	5.0
16	30.0	57.0	13.6
17	59.2	30.0	10.8

All values are expressed as % *w*/*w*.

**Table 2:** Miscibility and solubilization capacity of different surfactants with propylene glycol measured at (23 ± 2 °C).

Surfactant	Miscibility with Propylene Glycol	Solubility of Candesartan Cilexetil (mg/g)
Span 80	Immiscible	-
Span 20	Immiscible	-
HCO-10	Immiscible	-
Tween 80	Miscible	12.57 ± 0.14
Tween 20	Miscible	12.09 ± 0.12
Kolliphor EL	Miscible	11.66 ± 0.09
HCO-60	Miscible	10.24 ± 0.03
HCO-30	Miscible	9.44 ± 0.22
Tween 85	Miscible	9.03 ± 0.26
Tween 60	Miscible	8.74 ± 0.16

Values represent mean ± standard deviation (n = 3).

**Table 3:** The measured response parameters for the suggested 17 surfactant-enriched cross-linked scaffold at atmospheric pressure (1 atm).

Run Number	Drug Loading (mg/g)	Onset of Solid-to-Liquid Transition (°C)	Duration of Solid-to-Liquid Transition (s)
1	13.3	31.5	95
2	14.4	35	189
3	15.3	33.5	149
4	11.1	33	166
5	11.2	30	83
6	16.6	36	200
7	18.7	34	91
8	13.6	33.5	144
9	10.8	30.5	84
10	12.2	33	136
11	12.5	32	96
12	18.2	36	178
13	16.2	35.5	203
14	14.3	33.5	149
15	15.9	33.5	102
16	11.9	32.5	107
17	19.1	37	207

**Table 4:** Statistical validation parameters for the studied responses.

Response	R^2^	Adjusted R^2^	Predicted R^2^	Adequate Precision	F-Value	*p*-Value	Lack of Fit (*p*-Value)
Drug loading (mg/g)	0.9270	0.9165	0.8905	25.46	88.84	<0.0001	0.3312
Onset of solid-to-liquid transition (°C)	0.9576	0.9515	0.9332	33.99	157.99	<0.0001	0.3922
Duration of solid-to-liquid transition (Sec)	0.8418	0.8192	0.7324	15.81	37.25	<0.0001	0.6761

**Table 5:** Component effects analysis of mixture design on measured responses.

Response	Tween 80	Propylene Glycol	Pluronic F68
Drug loading	+6.61 ***	−8.60 ***	+1.28 *
Onset of solid-to-liquid transition	+1.89 ***	−6.89 ***	+3.22 ***
Duration of solid-to-liquid transition	−5.08 ^ns^	−107.93 ***	+97.54 ***

Statistical significance determined by ANOVA (α = 0.05). *** *p* < 0.001; * *p* < 0.05; ns = not significant (*p* ≥ 0.05).

**Table 6:** Validation of optimized surfactant-enriched cross-linked scaffold formulation showing experimental values along with low and high 95% prediction intervals.

Response	95% PI Low	Data Mean	95% PI High
Drug loading	17.1	19.3	19.5
Onset of solid-to-liquid transition	35.3	35.5	36.7
Duration of solid-to-liquid transition	145.3	188.0	204.7

**Table 7:** Initial dissolution rate and dissolution efficiency of candesartan cilexetil powder, physical mixture, and surfactant-enriched cross-linked scaffold.

Test Agent	Mean	
	IDR (%/min)	DE (%)
Candesartan cilexetil powder	0.00 ± 0.0	1.78 ± 0.8
Physical mixture	2.20 ± 0.2	37.19 ± 4.9
Surfactant-enriched cross-linked scaffold	6.30 ± 0.4	92.87 ± 4.0

Values represent mean ± standard deviation (n = 3).

## Data Availability

The original contributions presented in this study are included in the article. Further inquiries can be directed to the corresponding authors.
